# 12 Months Results of Bond Strength and Endogenous Enzymatic Activity of Radicular Dentin Obturated With Bioceramic Sealer

**DOI:** 10.3290/j.jad.c_2128

**Published:** 2025-07-01

**Authors:** Allegra Comba, Jessica Giannatiempo, Andrea Dirutigliano, Andrea Baldi, Mario Alovisi, Nicola Scotti, Annalisa Mazzoni, Lorenzo Breschi, Leila Es Sebar, Damiano Pasqualini

**Affiliations:** a Allegra Comba DDS, PhD, Researcher, Department of Surgical Sciences, University of Turin, Italy. Writing (original draft).; b Jessica Giannatiempo DDS, Department of Surgical Sciences, University of Turin, Italy. Writing (original draft).; c Andrea Dirutigliano DDS, Department of Surgical Sciences, University of Turin, Italy. Sample preparation, methodology.; d Andrea Baldi DDS, PhD, Research Fellow, Department of Surgical Sciences, University of Turin, Italy. Methodology.; e Mario Alovisi DDS, PhD, Associate Professor, Department of Surgical Sciences, University of Turin, Italy. Data analysis.; f Nicola Scotti DDS, PhD, Full Professor, Department of Surgical Sciences, University of Turin, Italy. Supervision.; g Annalisa Mazzoni DDS, PhD, Full Professor, Department of Biomedical and Neuromotor Sciences, DIBINEM, University of Bologna, Alma Mater Studiorum, Bologna, Bologna, Italy. Supervision.; h Lorenzo Breschi DDS, PhD, Full Professor, Department of Biomedical and Neuromotor Sciences, DIBINEM, University of Bologna, Alma Mater Studiorum, Bologna, Bologna, Italy. Supervision, conceptualization.; i Leila Es Sebar PhD, Research Fellow, Politecnico di Torino, Turin, Italy. Data analysis.; j Damiano Pasqualini DDS, PhD, Associate Professor, Department of Surgical Sciences, University of Turin, Italy. Project administration, validation.

**Keywords:** bioceramic, bond strength, matrix metalloproteinases, push-out, in-situ zymography

## Abstract

**Purpose:**

Evaluation of radicular bond strength and dentinal matrix metalloproteinases (MMPs) activity with different endodontic sealers (traditional vs bioceramic), filling techniques (warm vs cold), and adhesive protocols (self-etch vs etch-and-rinse), after 24 hours and after one year (T0 vs T1).

**Materials and Methods:**

96 extracted, caries-free, single-rooted teeth were selected and shaped with Proglider, ProTaper Next X1-X2. Samples were randomly divided into four groups: warm filling with ZOE sealer; cold filling with resin-based sealer; cold filling with bioceramic sealer; warm filling with bioceramic sealer. After 7 days, a 10 mm post space was prepared using dedicated drills, and each group was divided into two subgroups according to the adhesive procedure (self-etch vs etch-and-rinse, SE vs ER) employed for fiber post cementation with dual resin cement. Samples were analyzed with push-out tests at T0 and T1. 16 additional non-carious multirooted teeth were prepared following the described groups and subgroups for *in-situ* zymography analysis at T0 and T1. A four-way ANOVA, post-hoc Tukey was used to test the four factors and one-way ANOVA to evaluate the differences within each variable (α = 0.05).

**Results:**

Bioceramic sealer showed significantly higher bond strength than traditional sealer (P <0.05), especially when associated with the warm filling technique. SE adhesive protocol performed significantly better (P <0.05) independently of the sealer used, the filling technique, and the aging time. Greater endogenous collagenolytic activity was identified within the hybrid layer of ER-treated samples compared to SE independently from the other variables tested. In addition, warm technique proved to significantly reduce MMPs activity compared to the cold technique.

**Conclusion:**

The results showed that bioceramic sealers should guarantee better results in radicular dentin bond strength, without altering the endogenous enzymatic activity. The heat produced during the root canal obturation might reduce the internal enzymatic activity but, in association with bioceramic sealers, after 12 months, it produces higher bond strength. Heat reduces the difference between the two adhesive systems. ER technique and aging increase enzymatic activity. Aging tends to increase bond strength, especially in traditional sealers groups associated with ER protocol.

The outcome assessment of endodontic therapy is critical for decision-making and treatment planning. Several studies have been published evaluating success and failure in endodontics, and it is generally accepted that endodontic therapy is a predictable procedure with an excellent long-term prognosis and a high tooth retention rate.^45^ The longevity of endodontically treated teeth depends on several factors, including the amount of remaining structure, the criteria for good root canal therapy, the control of pulp-space infection, and post-endodontic restoration.

Gutta-percha still is the core filling material of choice because of its dimensional stability and its ability to be condensed against the irregular walls of the canals.^57^ Textbook accounts describe various filling methods available with gutta-percha, including cold condensation techniques (single-cone technique and lateral condensation technique) and warm condensation techniques (vertical condensation, the continuous wave of obturation technique, and the carrier-based technique).^9,27,47,57^ Along with cleaning and shaping the root canal system, it has been assumed that three-dimensional obturation is a fundamental condition to obtain a hermetic seal in endodontically treated teeth. Since gutta-percha cannot bond to dentin walls, it requires the association with a root canal sealer to provide an effective seal.^30^

Different types of root canal sealers have been introduced in endodontics^39^: zinc oxide-eugenol sealers, epoxy-resin-based sealers, calcium hydroxide-based sealers, glass ionomer-based sealers, and, more recently, bioceramic sealers.

Because of their physicochemical and biological properties bioceramics have recently gained popularity in the modern practice of endodontics.^56^ Bioceramics are bioinert and biodegradable ceramic materials specifically designed for medicine and dentistry. They include hydroxyapatite, bioactive glass, resorbable calcium phosphates, alumina, zirconia, coatings, and composites.^55^

Endodontic bioceramics are dimensionally stable and not sensitive to moisture or blood contamination.^25^ Hard and insoluble when placed, they expand slightly on setting.^26^ They are biocompatible and bioactive; they also revealed antibacterial properties. With a pH setting above 12, when these materials make contact with tissue fluids, they release calcium hydroxide, which interacts with phosphates in the tissue fluids, forming hydroxyapatite.^44^

In the endodontic field, they found application as a material for pulp capping, pulpotomy, perforation repair, obturation of open apices, and as a sealer for root canal filling in association with gutta-percha.^21,54^ Initially, it was recommended to use them as sealers with a single-cone technique,^14^ but recent studies have shown that bioceramics could be used both with cold and warm obturation techniques.^22^

However, increasing knowledge of the outcome of endodontic therapy has demonstrated that even the best filling techniques and materials are not sufficient to provide a complete, permanent seal of the root canal system against leakage from the oral cavity. Clinical evidence suggested quite clearly that we need to include the quality of the coronal restoration as a criterion for success in endodontics.^48^ As shown by Ray and Trope, the success rate of treatment is correlated equally with the quality of the root filling and the quality of the post-endodontic restoration.^45^

Teeth that have undergone root canal treatment are usually structurally compromised. Due to the massive loss of coronal and/or radicular tissue, the rehabilitation of these teeth often requires intraradicular retainers.^4,8^ Fiberglass posts are commonly used for the restoration of endodontically treated teeth because of the similarity of their elastic modulus to that of dentin.^3^ The material of choice for the cementation of fiber-reinforced posts is resin cement along with adhesive systems to obtain the bonding to the root canal dentin.^37^ Used in etch-and-rinse (ER) or self-etch (SE) mode, the adhesive procedure is responsible for the formation of a hybrid layer and intratubular tags which result from the infiltration of resinous monomers within the exposed collagen fibrils.^28^

A stable bond between the fiber post and radicular dentin is one of the keys to achieving a long-term, successful post-endodontic adhesive restoration.^50^ Several chemical reactions due to the oral environment, combined with changes in the tooth structure, could adversely affect the durability of the adhesive interface.^3^ Despite the advancements in adhesive systems and the improvements in composite cement, the adhesive interface on the organic dentin phase is still susceptible to long-term degradation.^16^ Resin–dentin bond degeneration can happen due to the hydrolytic breakdown of the resin and denuded dentinal collagen fibrils of the hybrid layer induced by endogenous proteolytic enzymes such as matrix metalloproteinases (MMPs).^23,24^ Bonding failure can lead to the formation of micro gaps, easily penetrable by pathogens, and to the subsequent loss of retention of the fiberglass post.^7^ That means that successful post-retained adhesive restoration depends critically on adequate bond strength between the post and the dentinal surface, and the leakage, located both apically and coronally, may affect the final outcome of endodontically treated teeth.

Performing an adhesive bond is influenced not only by the root canal filling techniques and the type of endodontic sealer, but also by the adhesive procedure for post cementation.

Considering the clinical relevance of bond strength to dentin, the objective of the present *in-vitro* study was to evaluate at baseline (T0, 7 days) and over time (T1, 12 months) radicular bond strength and dentinal MMPs activity using different:

endodontic sealers (traditional vs bioceramic)
filling techniques (warm vs cold)
adhesive protocols (self-etch vs etch-and-rinse)

The null hypotheses tested were: 1) the type of endodontic sealer has no effect on the endogenous enzymatic activity within the hybrid layer and the radicular bond strength; 2) the filling technique has no effect on the endogenous enzymatic activity and the radicular bond strength; 3) the adhesive protocol has no effect on the endogenous enzymatic activity and the radicular bond strength; 4) there are no differences between the endogenous enzymatic activity and bond strength immediately and over time.

## MATERIALS AND METHODS

### Specimen Preparation

Extracted, caries-free, human single-rooted teeth, with similar radicular length and diameter, were used for the present study (mostly maxillary central incisors, maxillary lateral incisors, and maxillary single-rooted premolars). The specimens were stored in 0.5% chloramine at 4°C and used within 1 month after harvesting. After debriding the root surface, each tooth was sectioned at the cementoenamel junction, perpendicularly to the longitudinal axis of the tooth, to visualize canal morphology. Among all the collected teeth, ninety-six specimens were selected, each with a circular-shaped canal and at least 12 mm of root length. Root canal treatment was performed using ProGlider and ProTaper Next X1–X2 (Dentsply Sirona Maillefer, Ballaigues, Switzerland) to the working length. During instrumentation, the canals were irrigated with 10 ml of 5% sodium hypochlorite (Niclor 5; Ogna, Muggiò, Italy), alternated with 2 ml of 10% ethylenediamine tetra-acetic acid (Tubuliclean; Ogna), using a 2-ml syringe and a 22-gauge needle. The clean and shaped root canals were rechecked under 20× magnification using an optical microscope (OPMI ProErgo, Carl Zeiss, Germany) to confirm the shape of the coronal part of the canal after instrumentation and to exclude the presence of visible cracks (irregular, oval, and cracked canals were discarded). Canals that satisfied those criteria were randomly assigned to one of the following groups, according to the filling technique and the endodontic sealer used.

Group 1: samples obtured by a continuous wave of condensation technique, using Conform Fit Gutta-Percha for ProTaper Next Endodontic Files ×2 (Dentsply Sirona Maillefer, Ballaigues, Switzerland) and sealed with zinc oxide-eugenol cement (Pulp Canal Sealer EWT; Kerr, Sybron, Romulus, MI, USA)
Group 2: samples obtured by the single-cone technique, using Conform Fit Gutta-Percha for ProTaper Next Endodontic Files ×2 (Dentsply Sirona Maillefer, Ballaigues, Switzerland) and sealed with epoxy-resin-based cement (AH Plus; DENTSPLY DeTrey, Konstanz, Germany)
Group 3: samples obtured by the single-cone technique, using Gutta-Percha TotalFill® BC Points™ 0.6 (FKG Dentaire, Chaux-de-Fonds, Switzerland) and sealed with bioceramic sealer TotalFill BC Sealer (FKG Dentaire, Chaux-de-Fonds, Switzerland)
Group 4: samples obtured by a continuous wave of condensation technique, using Gutta-Percha TotalFill® BC Point™ 0.6 (FKG Dentaire, Chaux-de-Fonds, Switzerland) and sealed with bioceramic sealer TotalFill® BC Sealer HiFlow™ (FKG Dentaire, Chaux-de-Fonds, Switzerland).

Each root-treated tooth was placed in a 100% relative humidity chamber at 37°C for 7 days to facilitate the setting of the sealer.

### Fiber Post Cementation

A 10 mm post space was subsequently created in each root-filled canal using fiber post drills (RelyX Fiber Post, 3M ESPE, St. Paul, MN, USA). Before luting, the correct length of each fiber post (RelyX Fiber Post, Size 2) was verified. Each fiber post was cleaned in ethanol for 30 s before the application of a silane coupling agent (Ceramic Primer; 3M ESPE, St. Paul, MN, USA). The primer-coupled fiber post was air-dried for 5 s.

The specimens were randomly divided into two subgroups according to the adhesive protocol employed:

Subgroup 1: samples treated with universal bonding (Clearfil Universal Bond Quick, Kuraray Noritake, Tokyo, Japan) used in an SE mode and fiber post-luted with a dual-cure resin luting cement (DC Core Automix, Kuraray Noritake, Tokyo, Japan) used according to the manufacturer’s instructions and inserted into the post space with a suitable-sized mixing tip;
Subgroup 2: samples treated with universal bonding (Clearfil Universal Bond Quick, Kuraray Noritake, Tokyo, Japan) used in an ER mode and fiber post-luted with a dual-cure resin luting cement (DC Core Automix, Kuraray Noritake, Tokyo, Japan) used according to the manufacturer’s instructions and inserted into the post space with a suitable-sized mixing tip.

After the post was inserted into the canal space for 1 min, the luting cement was light-cured with a light-emitted diode curing light (Translux Power Blue, Heraeus Kulzer, Hanau, Germany). Light curing was performed for 40 s each from the cervical surface of the root in the direction of the longitudinal axis, and then obliquely from the buccal and palatal surfaces (total 120 s). After polymerization, the post-luted specimens were stored in distilled water at 37°C for 24 h. Six 1-mm thick slices were prepared from each specimen using a low-speed diamond saw (Micromet, Remet, Bologna, Italy) using water cooling. A mark was placed on the coronal side of each section with an indelible marker. The marked specimens were stored in artificial saliva at 37°C.^42^

### Resistance of Fiber Post to Dislodgement

A micro push-out test was used to evaluate the ability of the fiber posts to resist dislodgement from the bonded canal walls. Testing was conducted after 24 h and after one year of storage in artificial saliva. Push-out was performed by applying an axial load to the post at a crosshead speed of 0.5 mm/min, using an Instron Machine I model 10/D (MTS, Eden Prairie, MN, USA). The apical surface was placed facing the punch tip, ensuring that loading forces were introduced from an apical to a coronal direction. Bond failure was manifested by the dislodgment of the fiber post from the root section. Push-out strength data were converted to MegaPascals (MPa) by dividing the load in Newtons by the bonded surface area (SL) in mm^2^, and SL was calculated as the lateral surface area of a truncated cone using the formula:

SL = (π * (R+r)) * ((h^2^ + (R-r))^2^)^0.5^

where R is the coronal radius of the canal with the post, r the apical radius and h the thickness of the slice. The wider and the narrowest diameters were digitally measured using ImageJ software on a picture of the slice taken on a millimeter paper to set the scale, while the thickness of the slice was individually measured using a pair of digital calipers with 0.01 mm accuracy.

A single observer evaluated the debonded specimens using a stereomicroscope at 40x magnification. Failure modes were classified as: adhesive failure between dentin and cement (AD), adhesive failure between the cement and post (AP), cohesive failure within the cement (CC), cohesive failure within the post (CP), and mixed failure (M). The percentage of each type of failure mode within each group was calculated.

### In-situ Zymography of the Hybrid Layer

Sixteen additional non-carious human multirooted teeth, extracted for orthodontic reasons, were used for *in-situ* zymography. Crown removal, root canal treatment, obturation, and fiber post cementation were performed in the manner described in previous sections.

Six 1-mm thick slices were prepared from each specimen using a low-speed diamond saw (Micromet, Remet, Bologna, Italy) using water cooling. Each section was fixed to a glass slide using glue and polished with 4000-grit silicon carbide papers with water cooling to obtain specimens with a final thickness of ~ 50 μm. Testing was conducted after 24 h and after 1 year of storage in artificial saliva.

*In-situ* zymography was performed with self-quenched fluorescein-conjugated gelatin as the MMP substrate (E-12055, Molecular Probes, Eugene, OR, USA).^33^ The fluorescent gelatin mixture was placed on top of each slab and covered with a glass cover slip. Each glass slide was protected from light and incubated in a humidified chamber at 37°C for 24 h. Hydrolysis of quenched fluorescein-conjugated gelatin within the hybrid layer, indicative of endogenous gelatinolytic enzyme activity, was evaluated by examination of the glass slides with a multi-photon confocal laser scanning microscope (TCS SP5-AOBS 5-channel, Leica Microsystems, Buffalo Grove, IL, USA), using an excitation wavelength of 495 nm and an emission wavelength of 515 nm. Images were acquired using an HCX PL APO 40 ×/1.25 NA oil immersion objective always maintaining the same microscope setting. Optical sections (350 nm thick) were acquired from different focal planes. The stacked images were analyzed, quantified, and processed with ImageJ software (National Institute of Health, Bethesda, MD, USA). The fluorescence intensity emitted by the hydrolyzed fluorescein-conjugated gelatin was quantified and the amount of gelatinolytic activity assessed through the green signal within the hybrid layer was expressed in arbitrary units.

### Statistical Analysis

After checking the normality (Shapiro-Wilk test) and homoscedastic (modified Levene’s test) assumptions of the data sets, a four-way ANOVA and a post-hoc Tukey’s test were performed to investigate the effects of the four variables (“endodontic sealer,” “adhesive protocol,” “filling technique,” “aging”).

The Chi-square test was used to analyze differences in the failure modes. For all tests, statistical significance was preset at α = 0.05.

## RESULTS

Push-out bond strength data at T0 and T1 are expressed as means and standard deviations (MPa) and summarized in Table 1.

**Table 1:** Mean and standard deviation of push-out bond strength values (MPa) at T0 and T1 obtained for radicular dentin obturated with traditional and bioceramic sealer associated with warm and cold technique

	T0	T1
Traditional sealer/Cold SE	11.09 ± 8.36 ^a,A^	11.43 ± 5.23 ^a,A^
Traditional sealer/Cold ER	6.5 ± 3.12 ^b,A^	8.06 ± 5.11 ^b,A^
Traditional sealer/Warm SE	9.62 ± 4.89 ^b,A^	8.37 ± 3.37 ^b,A^
Traditional sealer/Warm ER	6.63 ± 3.05 ^b,A^	9.47 ± 4.15 ^b,B^
Bioceramic sealer/Cold SE	13.3 ± 9.04 ^a,A^	8.49 ± 5.48 ^b,B^
Bioceramic sealer/Cold ER	11.91 ± 9.36 ^a,A^	9.11 ± 4.23 ^b,A^
Bioceramic sealer/Warm SE	12.67 ± 8.09 ^a,A^	12.35 ± 9.65 ^a,A^
Bioceramic sealer/Warm ER	11.79 ± 4.31 ^a,A^	11.91 ± 7.44 ^a,A^
Different superscript lowercase letters indicate differences (P <0.05) within the columns. Different superscript uppercase letters indicate differences (P <0.05) within the rows.

Statistical analysis showed a significant difference for the factors “filling material” and “adhesive system.” The factors “filling technique” and “time” did not influence bond strength of treated samples.

More specifically, bioceramic sealer showed significantly higher bond strength than traditional sealer (P <0.05), especially when associated with the warm filling technique.

SE adhesive protocol performed significantly better (P <0.05) independently from the sealer used the filling technique and the aging time.

**Fig 1:**
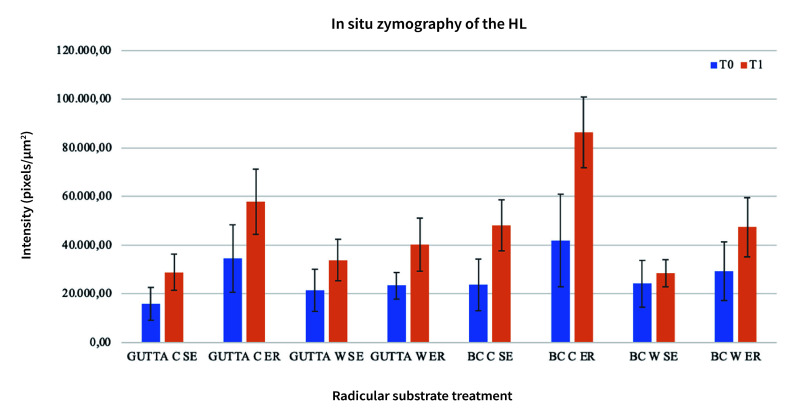
Graph showing mean and standard deviation of gelatinolytic activity, expressed as the intensity of green fluorescence (pixels/m^2^) within the hybrid layers (HL) at T0 and T1 obtained for radicular dentin obturated with traditional and bioceramic sealer associated with warm and cold technique.

**Fig 2:**
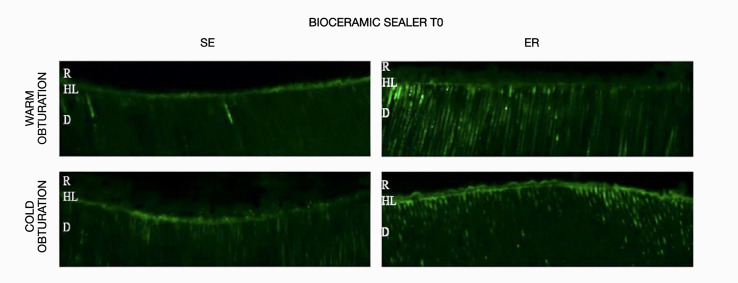
Representative confocal laser scanning microscopy images of resin-bonded radicular dentin interfaces of bioceramic sealer at T0 associated with warm and cold obturation techniques incubated with quenched fluorescein-labeled gelatin. Displayed image was acquired in the green channel. The green fluorescence represented areas with intense endogenous gelationolytic activity within the dentinal tubules and the hybrid layer.Abbreviations: D, dentin; HL, hybrid layer; R, composite resin.

**Fig 3:**
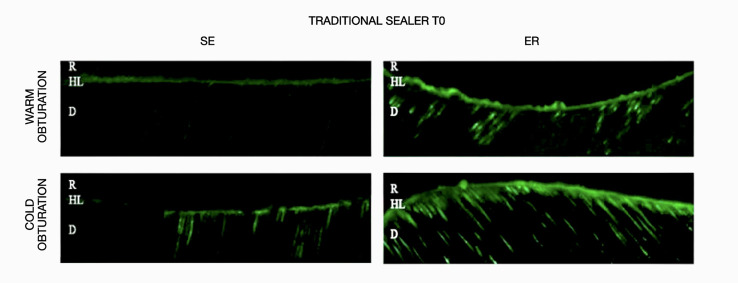
Representative confocal laser scanning microscopy images of resin-bonded radicular dentin interfaces of traditional sealer at T0 associated with warm and cold obturation techniques incubated with quenched fluorescein-labeled gelatin. Displayed image was acquired in the green channel. The green fluorescence represented areas with intense endogenous gelationolytic activity within the dentinal tubules and the hybrid layer.Abbreviations: D, dentin; HL, hybrid layer; R, composite resin.

**Fig 4:**
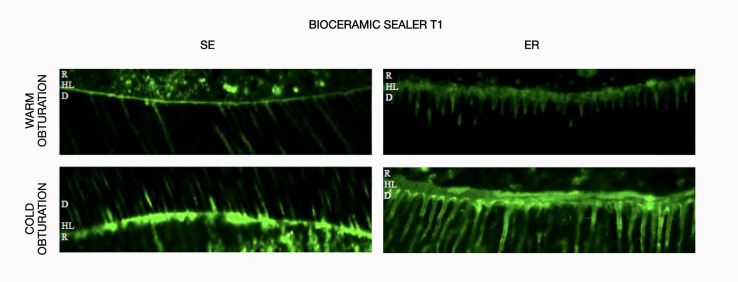
Representative confocal laser scanning microscopy images of resin-bonded radicular dentin interfaces of bioceramic sealer at T1 associated with warm and cold obturation techniques incubated with quenched fluorescein-labeled gelatin. Displayed image was acquired in the green channel. The green fluorescence represented areas with intense endogenous gelationolytic activity within the dentinal tubules and the hybrid layer.Abbreviations: D, dentin; HL, hybrid layer; R, composite resin.

**Fig 5:**
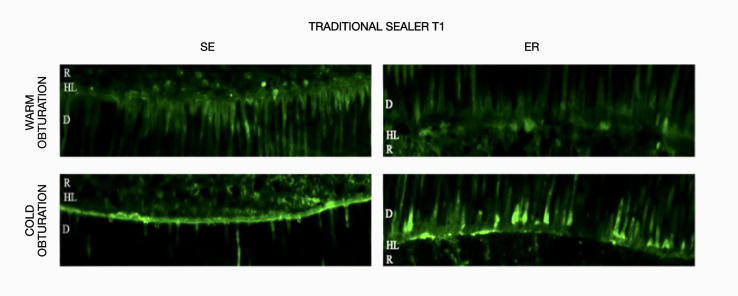
Representative confocal laser scanning microscopy images of resin-bonded radicular dentin interfaces of traditional sealer at T1 associated with warm and cold obturation techniques incubated with quenched fluorescein-labeled gelatin. Displayed image was acquired in the green channel. The green fluorescence represented areas with intense endogenous gelationolytic activity within the dentinal tubules and the hybrid layer.Abbreviations: D, dentin; HL, hybrid layer; R, composite resin.

The results obtained for *in-situ* zymography analysis at T0 and T1 are presented in Table 2 and Figure 1. Resin-bonded radicular dentin interfaces at T0 and T1 are shown in Figures 2, 3, 4, and 5, and revealed the most intense enzymatic activity within the dentinal tubules and the hybrid layer.

**Table 2:** Mean and standard deviation of gelatinolytic activity, expressed as the intensity of green fluorescence (pixels/m^2^) within the hybrid layers (HL) at T0 and T1 obtained for radicular dentin obturated with traditional and bioceramic sealer associated with warm and cold technique

	T0	T1
Traditional sealer/Cold SE	15.839.18 ± 6.831.67 ^a,A^	28.844.91 ± 7.360.86 ^a,B^
Traditional sealer/Cold ER	34.536.45 ± 13.919.14 ^b,A^	57.795.50 ± 13.453.53 ^a,B^
Traditional sealer/Warm SE	21.430.32 ± 8.708.22 ^a,A^	33.893.13 ± 8.444.89 ^a,B^
Traditional sealer/Warm ER	23.314.99 ± 5.473.26 ^a,A^	40.218.21 ± 11.042.27 ^a,B^
Bioceramic sealer/Cold SE	23.765.81 ± 10.573.34 ^a,A^	48.144.58 ± 10.442.90 ^a,B^
Bioceramic sealer/Cold ER	41.890.52 ± 18.912.93 ^c,A^	86.488.83 ± 14.567.36 ^a,B^
Bioceramic sealer/Warm SE	24.207.05 ± 9.635.44 ^a,A^	28.516.61 ± 5.653.18 ^a,A^
Bioceramic sealer/Warm ER	29.373.91 ± 12.072.13 ^b,A^	47.416.48 ± 12.122.74 ^a,B^
Different superscript lower case letters indicate differences (P <0.05) within the columns. Different superscript upper-case letters indicate differences (P <0.05) within the rows.

Failure mode analysis revealed that the predominant failure mode was adhesive between dentin and cement (AD) and between the cement and post (AP) as shown in (Table 3).

**Table 3:** Mode of failure at T0 and T1 obtained for radicular dentin obturated with traditional and bioceramic sealer associated with warm and cold technique

	T0	T1
AD	AP	CC	CP	M	AD	AP	CC	CP	M
Traditional sealer/Cold SE	46%	27%	5%	13%	9%	64%	19%	8%	8%	6%
Traditional sealer/Cold ER	58%	11%	12%	9%	10%	71%	3%	4%	16%	6%
Traditional sealer/Warm SE	53%	27%	2%	1%	13%	64%	10%	14%	10%	2%
Traditional sealer/Warm ER	17%	34%	15%	9%	5%	55%	9%	14%	12%	10%
Bioceramic sealer/Cold SE	36%	47%	16%	5%	15%	47%	25%	5%	18%	5%
Bioceramic sealer/Cold ER	24%	33%	6%	6%	19%	72%	8%	4%	7%	9%
Bioceramic sealer/Warm SE	80%	9%	2%	3%	6%	50%	15%	22%	4%	9%
Bioceramic sealer/Warm ER	46%	21%	6%	5%	27%	36%	28%	8%	11%	17%
The percentages of slices demonstrating adhesive failure between dentine and cement (AD), adhesive failure between the cement and post (AP), cohesive failure within the cement (CC), cohesive failure within the post (CP), and mixed failure (M) are reported

Statistical analysis for MMPs activity evaluation showed significant differences for the factors “filling technique,” “adhesive system,” and “time.” The variable “endodontic sealer” was not statistically significant.

More specifically, greater endogenous collagenolytic activity was identified within the hybrid layer of ER-treated samples compared to SE independently from the other variables tested. In addition, warm technique proved to significantly reduce MMPs activity compared to cold technique.

No fluorescence was detected in negative control groups prepared with non-specific inhibitors, including 1) EDTA-treated, 2) specimens incubated with 2 mM 1,10-phenanthroline, or 3) with standard nonfluorescent gelatin (data not shown).

## DISCUSSION

Over the years, new techniques and materials have been developed to improve the potential for healing and retention of endodontically treated teeth. Friedman et al demonstrated that the chance of endodontically treated teeth remaining functional over time is 91% to 97%.^18^

It is clinically accepted that this success rate depends on several elements, related both to the quality of the root canal treatment and the quality of the coronal restoration.

The present *in-vitro* study aimed to investigate the influence of various clinical factors, pertaining to both aspects, on the radicular bond strength and the endogenous enzymatic activity at baseline and after artificial aging.

In the attempt to create a better interface between root canal walls and sealer, coronal and apical leakages of obtured canals have been increasingly investigated. Several studies have shown that resin–dentin bond strength decreases over time due to the loss of the hybrid layer integrity.^29^ The degradation of the area of adhesion formed by dentin collagen matrix and resin adhesive is related to the activity of endogenous collagenolytic enzymes like matrix metalloproteinases (MMPs).^52^ Bond strength can be evaluated through several techniques such as shear strength, micro tensile, pull-out, or push-out tests.^5^ In accordance with other relevant studies, the push-out test was the method selected.^2,20,36^

For many years it has been questioned which is the most appropriate endodontic sealer to fill the root canal system. Recently, the exclusive use of traditional sealers was combined with the use of bioceramics. Bioceramic sealers can bond to dentin by a process known as alkaline etching, which allows ions exchange where the minerals of bioceramic sealer permeate the dentin and develop a mineral infiltration zone at the dentin-sealer interface.^13^

Our results are in agreement with previous studies which have demonstrated that bioceramic sealers showed similar and higher bond strength when compared with eugenol-based sealers and resin-based sealers.^41^ In our case, samples obtured with bioceramic sealers displayed a significantly higher bond strength to root dentin both at baseline and after 12 months.

Even if some authors concluded that the obturation technique did not significantly affect the bond longevity of the adhesive interface in canals obtured with a bioceramic sealer,^1^ in the present study, the effect of the filling method became evident over time. Samples sealed with bioceramic associated with the single-cone technique have shown lower bond strength values after artificial aging than samples obtured with bioceramic combined with the warm condensation technique.

Achieving durable resin–dentin bonds is still challenging for contemporary adhesive dentistry. Regardless of enhancements in dental materials,^10^ the adhesive–dentin interface within the hybrid layer created by actual dentin-bonding systems may lose strength over time.^53^ The capacity of the adhesive procedures to increase the collagenolytic activity of endogenous dentinal matrix metalloproteinases (MMPs) is now recognized.^32^ Irrespective of the ER or SE method employed, the acidity of these resin monomers probably contributes to the activation process of the MMPs exposed during adhesive steps.^34^ In the present study, dentin bonding was accomplished using ER and SE modes to investigate the effects of different bonding agents on radicular bond strength.

According to the push-out test, using a self-etch adhesive resulted in significantly higher bond strength values in samples obtured with traditional sealer and both filling techniques at baseline. Despite some studies disagreeing,^19^ several are in accordance with this outcome.^6,31,47,49,59^ Interestingly, results after 12 months suggested that heat application seems to reduce the discrepancy related to the adhesive systems. SE group performed better in samples obtured with traditional sealer and cold filling technique; samples obtured with warm filling technique showed no significant difference between ER group and SE group.

In agreement with other publications,^11,17,34^ the screening of the intrinsic proteolytic activity in adhesive-treated dentin was performed with *in-situ* zymography, which enabled precise localization of MMPs within the hybrid layer.^33^ Previous research detected the effect of the obturation technique in association with several sealers launched in the market on endogenous enzymatic activity.^12^ The present study represents the first attempt to include a new type of premixed bioceramic sealer composed of calcium silicate, calcium hydroxide, zirconium oxide, and calcium phosphate. Several authors supported the benefits of these materials in coronal dentin, revealing a decreased collagenolytic activity probably related to their bioactivity.^40^ However, according to the results obtained, no differences on the endogenous enzymatic activity within the hybrid layer emerged between groups. This could be attributed to the structural differences between coronal and intraradicular dentin and to endodontic-related procedures, including obturation.

The variation of the temperature on the root canal surface during the warm condensation technique and its effect on periradicular tissues have been extensively studied.^35^ However, to the authors’ best knowledge, no previous study has evaluated the effect of the temperature on the MMPs activity inside radicular dentin. Regardless of the type of sealer, data showed a decreased collagenolytic activity with the warm condensation technique. Many factors are responsible for the folding and stability of MMPs’ endogenous proteins. This result may be explained by a mechanism of thermal denaturation of the protein component, but further research is needed.

According to dental literature, the value of the enzymatic activity inside the root canal may vary, depending on the adhesive protocol.^15,38^ In the present study, a universal adhesive system was tested in self-etch and etch-and-rinse modes to evaluate the clinical relevance of such changes.

Regardless of the sealer and the root-filling technique, the ER protocol revealed that acid-etched dentin resulted in significantly more extensive proteolytic activity within the hybrid layer when compared to the application of the same adhesive in SE mode.

Furthermore, irrespective of the sealer, the root-filling technique, and regardless of whether dentin is exposed to etch-and-rinse or self-etch adhesive protocol, data revealed an increased proteolytic activity after 12 months in accordance with previous studies on coronal and radicular dentin.^51^ Indeed, once adhesive procedures are performed, MMPs inside the hybrid layer start their collagenolytic activity that continues over time.

## CONCLUSIONS

Within the limits of the present study, it can be concluded that using bioceramic sealers resulted in higher radicular bond strength, especially when associated with the warm condensation technique over time.

In addition, considering the endogenous enzymatic activity, bioceramic sealers may represent a good alternative to traditional sealers as no alteration were detected both at baseline and over time.

The multi-mode adhesive system showed that acid-etched dentin resulted in significantly more extensive proteolytic activity. It seems that the heat application might decrease the endogenous proteolytic activity, but the mechanism of this interaction is still unclear.

More studies are, anyway, needed to evaluate the influence of temperature variation inside the root canal on proteolytic activity, in addition to clinical studies to verify the behavior of these materials under the influence of the oral environment.
